# Predicting Major Bleeding in Native Kidney Biopsies: From External Validation of the Universal Bleeding Score to a New Clinical Tool

**DOI:** 10.3390/jcm15176515

**Published:** 2026-08-23

**Authors:** Andreea Niculescu, Gabriel Ștefan, Simona Stancu, Otilia Ciurea, Simona Cinca, Adrian Zugravu, Cristina Căpușă

**Affiliations:** 1 Department of Nephrology, University of Medicine and Pharmacy “Carol Davila”, 020021 Bucharest, Romania; calenic.andreea@gmail.com (A.N.); simonastancu2003@yahoo.com (S.S.); otilia3091@yahoo.com (O.C.); adzugravu@yahoo.com (A.Z.); ccalexandr@yahoo.com (C.C.); 2 “Dr. Carol Davila” Teaching Hospital of Nephrology, 010731 Bucharest, Romania; simona_cinca@yahoo.com

**Keywords:** percutaneous native kidney biopsy, biopsy-related complications, haemorrhagic complications, haematoma, bleeding risk, risk score, prediction model

## Abstract

**Objectives**: A percutaneous native kidney biopsy remains the gold standard for diagnosing glomerular, tubulointerstitial and vascular kidney diseases. Although generally safe, it may cause major haemorrhagic complications. Using the French national registry, Kaczmarek et al. developed a simplified bleeding risk score (anaemia, female sex, heart failure, acute kidney injury; range: 0–5), achieving an AUC of 0.755 in native biopsies. We aimed to externally validate this score in a Romanian cohort and to assess whether additional clinically relevant predictors could improve discrimination for major bleeding. **Methods**: We conducted a retrospective cohort study of all consecutive adults undergoing an ultrasound-guided percutaneous native kidney biopsy at a tertiary nephrology centre in Romania between January 2008 and December 2024. The primary outcome was a composite major bleeding event: clinically significant haematoma or haemorrhage, blood transfusion, angiographic intervention or nephrectomy. The Kaczmarek score was validated using a receiver operating characteristic (ROC) analysis. Candidate predictors were assessed by multivariable logistic regression, and a simplified score was derived from the regression coefficients. **Results**: Among 3081 patients, 162 (5.3%) experienced major bleeding. The Kaczmarek score showed modest discrimination (AUC: 0.624, 95% CI: 0.585–0.663). In the multivariable analysis (*n* = 2506 complete cases), anaemia, heart failure, solid neoplasm and fibrinogen were independently associated with major bleeding. An eight-component score (anaemia, heart failure, solid neoplasm, and fibrinogen < 511 mg/dL; female sex, hypertension, liver disease, and eGFR < 30 mL/min/1.73 m^2^) achieved an AUC of 0.676 (95% CI: 0.631–0.717), outperforming the Kaczmarek score. **Conclusions**: The French score performed only modestly in our cohort. Adding comorbidities, kidney function and haemostatic parameters, particularly fibrinogen, improved the risk discrimination.

## 1. Introduction

A percutaneous native kidney biopsy remains the gold standard for the histological diagnosis of glomerular, tubulointerstitial and vascular kidney diseases. The procedure is generally safe; however, haemorrhagic complications—including clinically significant haematoma, blood transfusion, angiographic intervention and, rarely, nephrectomy—occur in 1–14% of patients, depending on the definition and population studied [1]. Beyond haemorrhagic morbidity, biopsy-related death—although exceedingly rare—has also been reported and represents the most severe end of the complication spectrum [2]. In the largest nationwide analysis to date, Halimi et al. reported major bleeding in 5% of 52,138 biopsies in France and demonstrated a twofold increase in the 30-day mortality associated with such events [2]. A subsequent study from the same group confirmed that the transjugular route reduced bleeding in high-risk patients identified by a pre-procedure score [3]. These data underscore the need for reliable pre-procedural risk stratification.

Several risk scores and prediction models have been proposed. Kaczmarek et al. developed a simplified universal score (anaemia, female sex, heart failure, acute kidney injury; range: 0–5) from the French national database, achieving an AUC of 0.755 in 55,026 native biopsies [4]. Schorr et al. created an online kidney biopsy risk calculator (KBRC) incorporating the age, BMI, platelet count, haemoglobin and kidney size (C-statistic: 0.83 in 617 biopsies) [5], later temporally validated in 400 additional biopsies [6]. Wang et al. proposed a logistic equation using the mean arterial pressure, bleeding time, eGFR, kidney length and cortical thickness (AUC: 0.848) [7], and Mejia-Vilet et al. developed a point-based score with an AUC of 0.872 [8]. Lim et al. focused on high-risk patients with a serum creatinine ≥ 2 mg/dL, identifying haemoglobin < 10 g/dL and platelets ≤ 216 × 10^9^/L as key predictors (AUC: 0.834) [9]. More recently, Li et al. demonstrated that adding post-biopsy ultrasound data to the KBRC improved the AUC from 0.683 to 0.867 [10], and Zheng et al. identified D-dimers and eGFR as independent predictors of severe bleeding in 2822 patients [11].

Despite this growing body of work, important gaps persist. First, the Kaczmarek universal score—the most practical tool for widespread clinical use—has not been externally validated. Second, the existing scores derived from administrative databases lack laboratory data, such as fibrinogen, a central mediator of secondary haemostasis that is frequently deranged in patients with kidney disease. Third, comorbidities known to affect coagulation—liver disease and solid neoplasm—have not been incorporated into simplified scoring systems.

In this study, we externally validated the Kaczmarek score in 3081 consecutive native kidney biopsies at a Romanian tertiary centre, derived a new clinical risk score incorporating fibrinogen and comorbidity data unavailable in the original administrative database, performed bootstrap internal validation, and compared the discriminative performance of the two scores.

## 2. Materials and Methods

### 2.1. Study Design and Population

This was a single-centre retrospective cohort study of all consecutive adult patients who underwent an ultrasound-guided percutaneous native kidney biopsy at a tertiary nephrology centre in Romania between January 2008 and December 2024. Clinical and laboratory data were extracted from electronic medical records. The study was conducted in accordance with the Declaration of Helsinki and approved by the institutional ethics committee.

### 2.2. Biopsy Procedure and Peri-Procedural Management

All biopsies were performed percutaneously under real-time ultrasound guidance using an automated spring-loaded 16- or 18-gauge needle, with two cores obtained in most patients. The procedures were performed by experienced nephrologists or by nephrology trainees under direct supervision. According to the institutional protocol, antiplatelet agents were discontinued at least seven days before the biopsy and oral anticoagulants were withheld before the procedure, with low-molecular-weight heparin bridging when clinically indicated; desmopressin was not routinely administered. After the biopsy, the patients remained supine for at least 6 h with a compression bandage, followed by overnight bed rest. Their vital signs were monitored every 15 min for 2 h, hourly for 4 h, and every 2 h thereafter; each void was inspected for macroscopic haematuria; and the haemoglobin was measured at 24 h or as needed. Protocol ultrasonography was performed immediately after the biopsy, at 6 h, and at 24 h, or as clinically indicated. The biopsy technique and peri-procedural management protocol remained standardised throughout the study period; however, the needle gauge, number of passes, cortical thickness and operator identity were not systematically recorded at the individual patient level.

### 2.3. Primary Outcome

The primary outcome was a composite major bleeding event, defined as any of: clinically significant haematoma or haemorrhage, blood transfusion, angiographic intervention or nephrectomy. Each patient was counted once regardless of the number of individual sub-events. This definition was harmonised with the criteria used by Kaczmarek et al. [4].

### 2.4. Methods for External Validation of the Kaczmarek Score

The Kaczmarek score was calculated using the original formula: anaemia = 1 point, female sex = 1, heart failure = 1, and acute kidney injury = 2 (range: 0–5). External validation assessed discrimination by the area under the receiver operating characteristic curve (AUC) with bootstrap 95% confidence intervals (2000 resamples). The calibration was evaluated by comparing the observed bleeding rates at each score level with those reported in the original native kidney biopsy cohort [4].

### 2.5. Candidate Predictors

Pre-procedural variables evaluated as candidate predictors included demographics (age, sex), comorbidities (hypertension, diabetes mellitus, obesity, heart failure, ischaemic heart disease, cerebrovascular disease, peripheral vascular disease, solid neoplasm, liver disease), haematological parameters (anaemia, defined as haemoglobin below sex-specific WHO thresholds; thrombocytopenia, defined as a platelet count < 150 × 10^3^/µL), kidney function (eGFR < 30 mL/min/1.73 m^2^ by CKD-EPI equation, acute kidney injury) and coagulation markers (fibrinogen, INR).

Both eGFR and fibrinogen were dichotomised for model development. For eGFR, a threshold of 30 mL/min/1.73 m^2^ was chosen based on previous literature linking impaired kidney function to uraemia-associated platelet dysfunction [1,9]. Fibrinogen was dichotomised at the cohort median (511 mg/dL), which showed a stronger association with the outcome than the conventional 300 mg/dL threshold or the continuous analysis.

### 2.6. Statistical Analysis

Continuous variables are reported as the median [25th–75th percentile] and compared using the Mann–Whitney U test. Categorical variables are reported as counts (percentages) and compared using the chi-squared test. Univariate binary logistic regression was performed for each candidate predictor, with results expressed as odds ratios (ORs) and 95% confidence intervals (CIs).

All candidate binary predictors were entered into a multivariable binary logistic regression model. Variables independently associated with major bleeding at *p* < 0.05 formed the core of the score. Additional variables were retained in the final score based on a consistent effect direction, clinical plausibility and established literature support, following the recommendation that predictor selection in clinical prediction models should integrate subject-matter knowledge to preserve clinical face validity and model stability, rather than relying solely on *p*-value thresholds [12,13,14].

A clinical risk score was derived using the regression-coefficient-based method [15,16]. Points were assigned proportionally to the β coefficients from the full multivariable model to preserve the adjustment for all candidate predictors: predictors with β > 0.50 received 2 points and those with β ≤ 0.50 received 1 point. Internal validation used bootstrap resampling (1000 iterations); optimism was subtracted from the apparent AUC to yield the optimism-corrected AUC. The comparative ROC analysis used bootstrap-derived 95% CIs and *p*-values (2000 resamples). The diagnostic performance was assessed at each cut-off; the optimal cut-off was identified by the Youden index. The patients were categorised into three risk groups: low (0–2 points), intermediate (3–4 points) and high (≥5 points). *p* < 0.05 (two-sided) was considered statistically significant. The analyses used R version 4.4.0 (R Foundation for Statistical Computing, Vienna, Austria) with the rms, pROC and boot packages.

## 3. Results

### 3.1. Study Population and Major Bleeding Rate

A total of 3081 patients were included (Table 1). The median age was 51 years [IQR: 39–63], 55.9% were male, and the median Charlson comorbidity index was 2 [1,2,3,4]. Hypertension was present in 66.4%, anaemia in 44.9%, heart failure in 8.2% and an eGFR < 30 mL/min/1.73 m^2^ in 32.0% of patients. A total of 192 patients (6.2%) were receiving haemodialysis at the time of the biopsy. Fibrinogen data were available for 2954 patients (95.9%; median: 511 mg/dL [IQR: 394–696]).

Major bleeding occurred in 162 of the 3081 patients (5.3%). The individual components were: haematoma/haemorrhage, 150 (4.9%); blood transfusion, 62 (2.0%); angiographic intervention, 25 (0.8%); and nephrectomy, five (0.2%). There was one biopsy-related death (0.03%). Patients with major bleeding had significantly higher rates of anaemia, heart failure, solid neoplasm, liver disease, a lower median haemoglobin, a lower median eGFR and a lower median fibrinogen (all *p* < 0.05; Table 1). The clinical indication for the biopsy (nephrotic syndrome, nephritic syndrome, nephrotic–nephritic syndrome, acute kidney injury, isolated urinary abnormalities or chronic kidney disease of undetermined aetiology) did not differ significantly between patients with and without major bleeding (*p* > 0.05).

### 3.2. External Validation of the Kaczmarek Score

**Figure 1:**
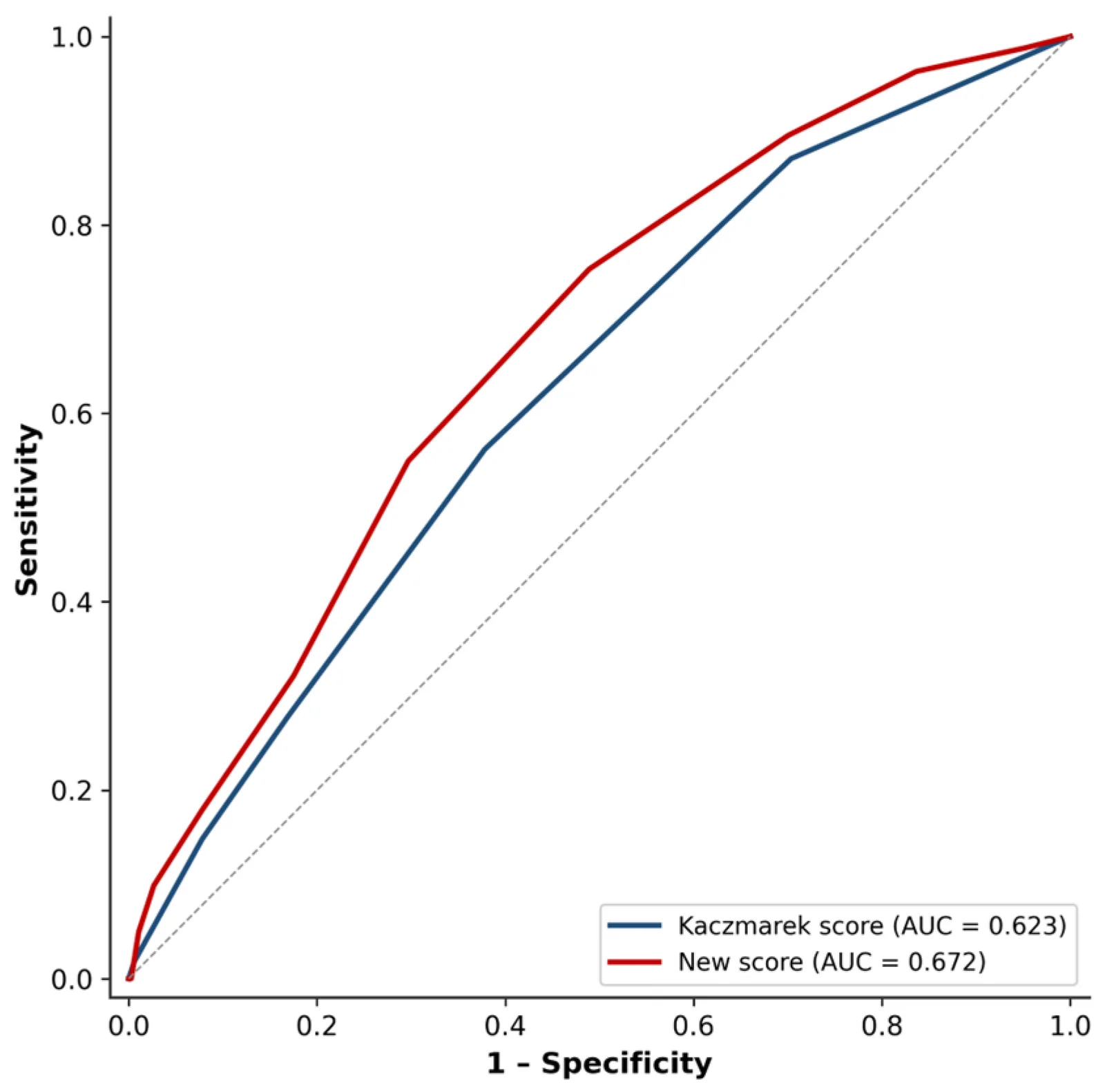
ROC curves comparing the Kaczmarek score (AUC = 0.624) and the new score (AUC = 0.676).

**Figure 2:**
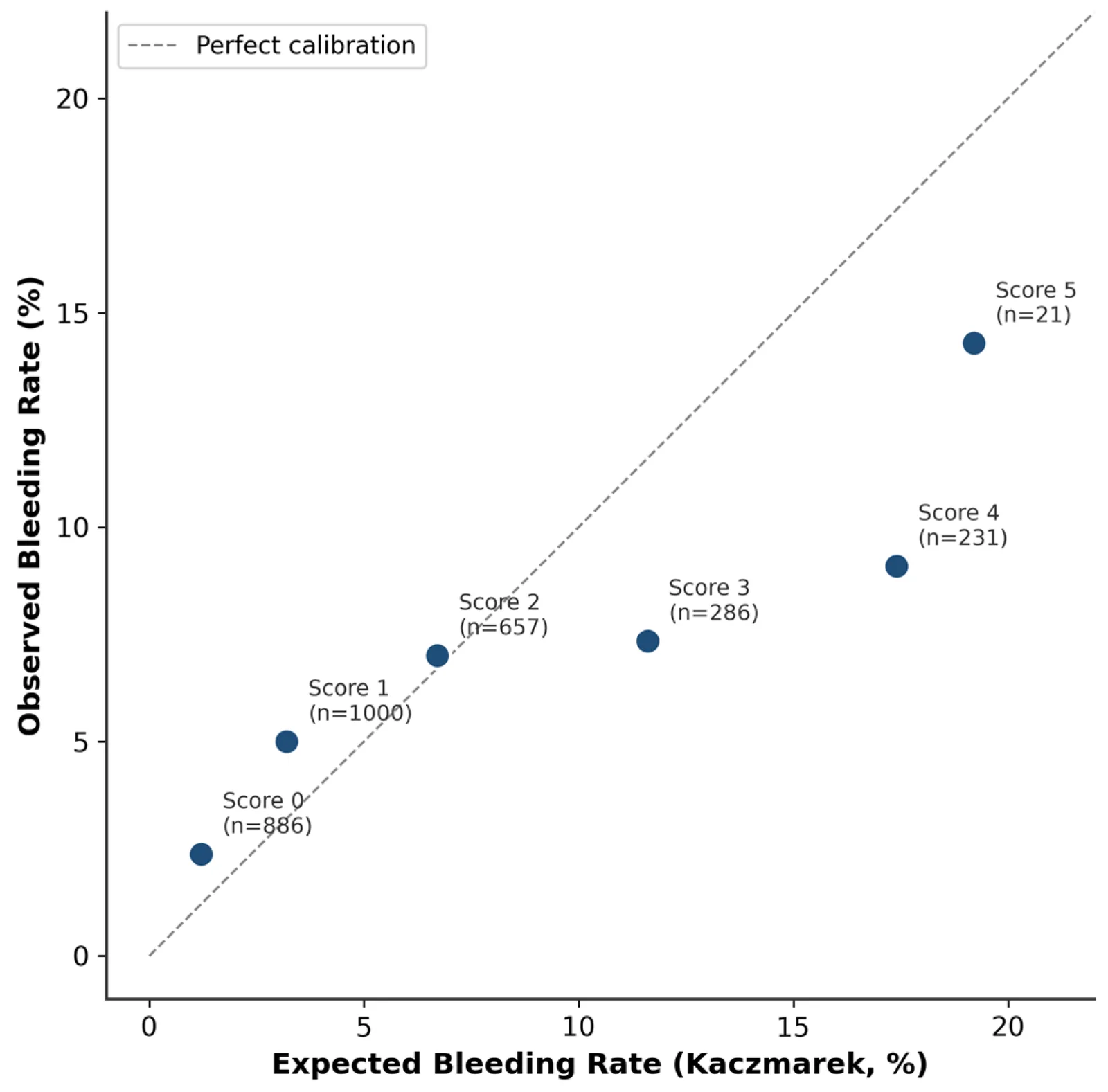
Calibration of the Kaczmarek score: observed vs. expected bleeding rates.

The Kaczmarek score showed modest discrimination, with an AUC of 0.624 (95% CI: 0.585–0.663) (Figure 1). The score demonstrated a consistent risk gradient: the observed bleeding rate increased from 2.4% for score 0 to 14.3% for score 5 (Table 2). The calibration analysis revealed that, at lower score levels (0–1), the observed rates exceeded the expected rates, while at higher levels (3–5), the observed rates were lower than expected, indicating an overestimation of the risk in the higher-risk strata of our population (Figure 2).

### 3.3. Univariate Analysis

In univariate logistic regression, the variables significantly associated with major bleeding in our cohort (*p* < 0.05) included anaemia, heart failure, solid neoplasm, cerebrovascular disease, eGFR < 30 mL/min/1.73 m^2^, liver disease, hypertension, acute kidney injury, fibrinogen < 511 mg/dL and female sex (Table 3). Of note, the platelet count, analysed as a continuous variable, was associated with major bleeding, whereas thrombocytopenia (<150 ×10^3^/µL) was not. This apparent discrepancy reflects the low prevalence of thrombocytopenia in a cohort selected for biopsy eligibility, which limited the statistical power for the dichotomised variable, together with the greater information retained by the continuous analysis; the per-unit odds ratio for the platelet count, although statistically significant, was of negligible magnitude, and neither variable was retained in the final score.

### 3.4. Multivariable Analysis and Score Derivation

In the multivariable model (*n* = 2506 complete cases), four variables were independently associated with major bleeding at *p* < 0.05: anaemia, fibrinogen < 511 mg/dL, heart failure and solid neoplasm. Four additional variables were retained in the final score based on consistent effect direction and clinical rationale [12,13,14]: female sex (OR: 1.41, *p* = 0.067), hypertension (OR: 1.46, *p* = 0.081), eGFR < 30 mL/min/1.73 m^2^ (OR: 1.10, *p* = 0.676) and liver disease (OR: 1.45, *p* = 0.152) (Table 4). The full multivariable model, including all 16 candidate predictors, is provided in Supplementary Table S1.

The resulting score comprised eight components with a maximum of 12 points (Table 4): anaemia (2), heart failure (2), solid neoplasm (2) and fibrinogen < 511 mg/dL (2) each received 2 points based on β coefficients > 0.50; female sex (1), hypertension (1), eGFR < 30 mL/min/1.73 m^2^ (1) and liver disease (1) each received 1 point.

### 3.5. Bleeding Rate by Score Level

**Figure 3:**
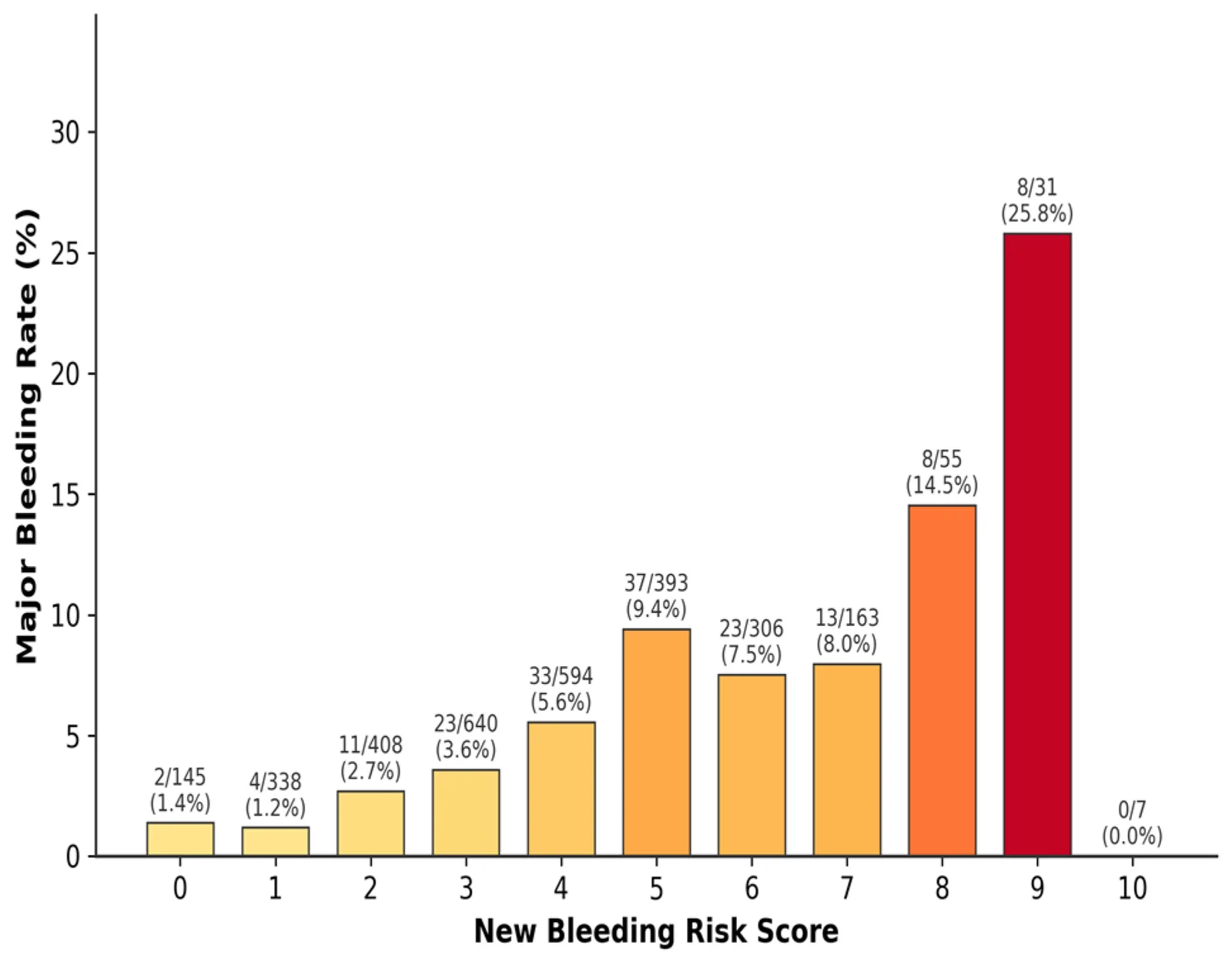
Observed major bleeding rate by new score level. Labels show events/total (rate).

**Figure 4:**
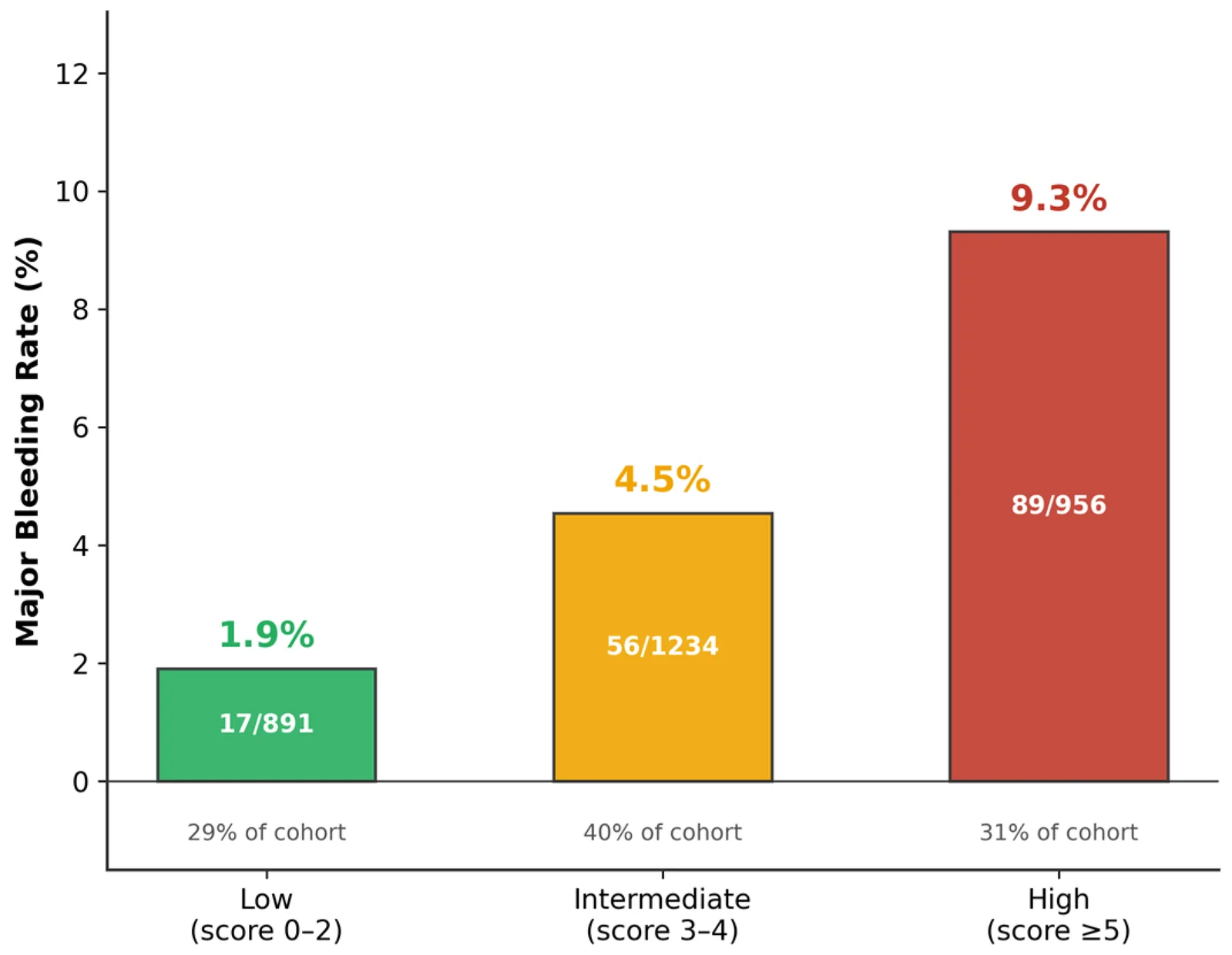
Major bleeding rate by risk category: low (0–2), intermediate (3–4), high (≥5).

The observed major bleeding rate showed a progressive gradient across score levels, from 1.0–1.5% at scores 0–1 to 16.7% at score 8 and 29.6% at score 9 (Figure 3, Table 5). When classified into three risk categories, the bleeding rate was 1.8% in the low-risk group (score: 0–2, *n* = 769, 28.4%), 4.6% in the intermediate-risk group (score: 3–4, *n* = 1079, 39.9%) and 9.3% in the high-risk group (score: ≥5, *n* = 857, 31.7%) (Figure 4). The fourfold increase from the low to the high category supports clinically meaningful risk stratification.

### 3.6. Discriminative Performance and Internal Validation

The new score demonstrated an AUC of 0.676 (95% CI: 0.631–0.717), significantly higher than the Kaczmarek score AUC of 0.624 (0.585–0.663) (ΔAUC = +0.052, 95% CI: +0.015 to +0.090, *p* = 0.004) (Figure 1). Bootstrap internal validation (1000 iterations) yielded an apparent AUC of 0.678 for the logistic model with a mean optimism of 0.018, resulting in an optimism-corrected AUC of 0.660. The small optimism estimate indicates limited overfitting.

The optimal cut-off was ≥4 points (Youden = 0.263), with a sensitivity of 75.0%, a specificity of 51.3%, a PPV of 8.0% and an NPV of 97.3% (Table 6). A higher threshold of ≥5 offered a greater specificity (69.7%) with a sensitivity of 55.6%, which may be more appropriate for identifying patients requiring extended post-procedural monitoring. The performance and classification characteristics of the new score compared with the Kaczmarek score are summarized in Table 7.

## 4. Discussion

In this single-centre retrospective study of 3081 percutaneous native kidney biopsies, we externally validated the French simplified score developed by Kaczmarek et al. [4] and derived a new eight-component clinical prediction score. Major bleeding occurred in 5.3% of patients, consistent with the 5% rate reported in the landmark French nationwide cohort [2] and the 5.1% major complication rate observed in the multicentre Italian ITA-KID-BIO registry [17]. The Kaczmarek score demonstrated modest discrimination in our cohort (AUC: 0.624), substantially lower than its reported performance in the original French dataset (AUC: 0.755 for native biopsies). Our newly derived score, incorporating fibrinogen below the cohort median, solid neoplasm, liver disease and eGFR < 30 mL/min/1.73 m^2^ alongside the original predictors, achieved significantly better discrimination (apparent AUC: 0.676; optimism-corrected AUC: 0.660; ΔAUC: +0.052, *p* = 0.004), with clinically meaningful risk stratification across three risk categories.

The attenuation in the Kaczmarek score performance upon external validation is a well-recognised phenomenon in clinical prediction research [12,13]. The original score was developed using administrative ICD-10 codes in a French nationwide database, and its high AUC in native biopsies (0.755) likely benefited from derivation-sample optimism and population-specific calibration [4]. In our cohort, the calibration analysis revealed that, at lower score levels, the observed rates exceeded expectations, while at higher levels, the score overestimated the risk, suggesting that the underlying case-mix in a Romanian tertiary centre differs meaningfully from the French reference population. A similar performance degradation has been observed with other kidney biopsy risk tools: the Schorr et al. KBRC, which achieved a C-statistic of 0.83 in its derivation cohort [5], showed good discrimination (C-statistic: 0.91) in a subsequent temporal validation at the same institution, but with only seven events and wide confidence intervals [6]. Li et al. reported an AUC of 0.683 for the base KBRC-5 model in a prospective Chinese cohort of 471 biopsies, which improved substantially only after incorporating post-biopsy ultrasound findings (AUC: 0.786 with immediate ultrasound, 0.867 with six-hour ultrasound) [10]. These findings collectively underscore that pre-procedural risk scores based solely on clinical variables face an inherent performance ceiling, and that population-specific recalibration is essential before clinical implementation.

A key contribution of the present study is the identification of fibrinogen as a clinically relevant predictor of post-biopsy bleeding. Fibrinogen below the cohort median (511 mg/dL) was independently associated with major bleeding (OR: 1.92, *p* < 0.001) and represented the strongest single addition to the Kaczmarek score framework. This finding is physiologically plausible: fibrinogen is central to secondary haemostasis, and lower levels—even within the normal range—may compromise clot formation at the biopsy site. The conventional laboratory threshold of 300 mg/dL failed to discriminate the risk in our population, likely because most patients referred for a kidney biopsy have elevated fibrinogen as an acute-phase reactant. The superiority of the cohort-median dichotomisation over the conventional deficiency threshold likely reflects the fact that, in a population enriched for glomerulonephritis, nephrotic syndrome and systemic inflammation, the absolute levels are shifted upward, and values considered normal may nevertheless reflect a relative depletion of the haemostatic reserve. To our knowledge, no previous kidney biopsy bleeding risk scores have incorporated fibrinogen, although coagulation parameters have been recognised as unmeasured confounders in large administrative studies [2,3]. Zheng et al. recently reported that D-dimers, another coagulation marker, independently predicted severe bleeding (OR: 1.27) in a Chinese cohort of 2822 patients [11], further supporting the value of incorporating haemostatic parameters into risk stratification.

Solid neoplasm was retained in the final score despite a low prevalence of 3.5%, based on its strong effect size (OR: 2.19, *p* = 0.023). Cancer was also identified as a risk factor in the original Halimi nationwide score, where it contributed 2 points [2]. Liver disease, similarly retained on clinical grounds (OR: 1.45), is a recognised cause of impaired coagulation factor synthesis and has been reported as an independent predictor in the ITA-KID-BIO prospective registry [17]. The inclusion of an eGFR < 30 mL/min/1.73 m^2^ is consistent with the extensive literature linking impaired kidney function to uraemic platelet dysfunction [1,9]. Lim et al., when studying 184 high-risk biopsies with a serum creatinine ≥ 2 mg/dL, found that the combination of an age ≥ 62 years, haemoglobin < 10 g/dL and platelets ≤ 216 × 10^9^/L predicted major bleeding with an AUC of 0.834 [9], reinforcing the importance of haematological and renal parameters. Zheng et al. similarly confirmed that a lower eGFR was independently associated with severe complications (OR: 0.90 per unit increase, *p* = 0.002) [11].

Placing our score in the broader landscape, the discriminative performance of pre-procedural scores has generally been moderate. The original Halimi nationwide score (19 diagnoses, Charlson and frailty indices) achieved an AUC of 0.80 in 52,138 patients [2], but is complex. The simplified Kaczmarek score (AUC: 0.755) [4] sacrificed discrimination for simplicity. Wang et al. achieved an AUC of 0.848 using the mean arterial pressure, bleeding time, eGFR, kidney length and cortical thickness [7], but this model requires measurements not routinely and consistently documented. The Mejia-Vilet score achieved the highest pre-biopsy AUC of 0.872, but has not been externally validated [8]. Our score, with an optimism-corrected AUC of 0.660, is based on eight readily available clinical and laboratory variables, including a marker of haemostatic reserve, without relying on complex composite indices, thereby facilitating reproducibility across clinical settings. The modest AUC reflects the fundamental challenge that major bleeding is also influenced by procedural factors such as the needle gauge, number of passes and operator experience, unavailable pre-procedurally [5,7,18].

Nevertheless, the score provides clinically useful risk stratification. The fourfold gradient from the low-risk (1.8%, score: 0–2) to the high-risk (9.3%, score: ≥5) category can inform shared decision-making regarding the biopsy indication, procedural route and monitoring intensity. Halimi et al. demonstrated that the transjugular route reduced bleeding in high-risk patients (OR: 0.83) [3], supporting risk-stratified procedural planning. Schorr et al. showed that a two-hour post-biopsy observation window was sufficient for outpatient biopsies [6], but higher-risk patients might benefit from extended monitoring. The negative predictive value exceeded 96% across all thresholds, confirming that low-scoring patients can be safely managed with standard protocols.

Li et al. demonstrated that incorporating post-biopsy imaging data improves the prediction substantially (AUC from 0.683 to 0.867) [10], but such information is unavailable at the time of the biopsy decision. Pre-procedural risk scores serve a different purpose: they identify patients who may benefit from procedural optimisation, the correction of modifiable risk factors, and more intensive post-procedural monitoring.

The strengths of this study include its large single-centre cohort with uniform biopsy protocols, the availability of coagulation data (fibrinogen, INR) absent from the administrative databases used by Halimi and Kaczmarek [2,4], rigorous bootstrap internal validation, and a direct head-to-head comparison with a validated external score.

Several limitations should be acknowledged. First, the single-centre retrospective design limits the generalisability; external validation in independent populations is needed. Second, the study relied on discharge diagnoses for comorbidity and outcome ascertainment, which may have resulted in misclassification—although this is similar to the methodology used by the French nationwide studies [2,3,4]. Third, procedural variables (needle gauge, number of passes, cortical thickness, operator experience, use of desmopressin) were not systematically recorded at the individual patient level, although the biopsy technique and peri-procedural management were standardised throughout the study period (Section 2.2). Wang et al. and Manno et al. have shown that procedural factors influence the bleeding risk [7,18], and their absence likely contributes to the moderate discrimination observed. Fourth, retaining four variables with *p* > 0.05 based on clinical plausibility, while supported by prediction-modelling guidelines [12,13,14], may be debated; however, their inclusion did not introduce meaningful overfitting (optimism: 0.018). Fifth, fibrinogen data were unavailable for 4.1% of the patients. Sixth, the full multivariable model with 16 candidate predictors yielded approximately eight events per variable (132 events ÷ 16 predictors), below the conventional minimum of 10 recommended for logistic regression [12]; however, the final score comprises only eight components (approximately 20 events per variable when applied to the full cohort), and bootstrap validation was employed to quantify and correct for any residual optimism. Seventh, the long recruitment period (2008–2024) is a potential source of bias: although the biopsy technique and peri-procedural protocol remained standardised (Section 2.2), ultrasound technology, operator expertise and post-procedural surveillance practices are likely to have evolved over time, and such temporal changes may have influenced both the observed bleeding incidence and the performance of the prediction model. Eighth, the score was deliberately restricted to pre-procedural variables available at the time of the biopsy decision; the clinical indication for the biopsy and the final histological diagnosis were therefore not incorporated, and in exploratory analyses, neither was associated with major bleeding in our cohort. Finally, this score was not designed for transplant kidney biopsies [4].

Future studies should pursue external validation in geographically diverse cohorts, assess the incremental value of post-biopsy ultrasound findings [10,11], and explore integration into electronic health records as a bedside calculator, similar to the KBRC [5].

## 5. Conclusions

External validation of the Kaczmarek universal bleeding risk score in a Romanian cohort revealed modest discrimination, substantially lower than in the derivation population. A newly derived eight-component score incorporating fibrinogen, solid neoplasm, liver disease and eGFR alongside established predictors significantly outperformed the Kaczmarek score and provided clinically meaningful risk stratification. While the overall discriminative performance remains moderate—a shared limitation of all pre-procedural bleeding risk tools—the score offers a practical framework for shared decision-making regarding the biopsy indication, procedural planning and post-procedural monitoring.

## Figures and Tables

**Table 1:** Baseline characteristics of the study population.

Variable	Total (*n* = 3081)	No Bleeding (*n* = 2919)	Bleeding (*n* = 162)	*p*
**Demographics**				
Age (years)	51 [39–63]	51 [39–63]	50 [39–64]	0.809
Male sex	1718 (55.9)	1641 (56.3)	77 (47.8)	0.035
Charlson comorbidity index	2 [1–4]	2 [1–4]	2 [1–4]	0.292
**Comorbidities**				
Hypertension	2042 (66.4)	1920 (65.9)	122 (75.3)	0.014
Diabetes mellitus	543 (17.7)	515 (17.7)	28 (17.3)	0.898
Obesity	675 (22.5)	646 (22.7)	29 (18.6)	0.226
Ischaemic heart disease	432 (14.1)	404 (13.9)	28 (17.4)	0.213
Heart failure	251 (8.2)	225 (7.8)	26 (16.0)	<0.001
Cerebrovascular disease	188 (6.1)	171 (5.9)	17 (10.5)	0.017
Peripheral vascular disease	269 (8.8)	260 (8.9)	9 (5.6)	0.144
Solid neoplasm	106 (3.5)	94 (3.3)	12 (7.5)	0.004
Liver disease	310 (10.1)	285 (9.8)	25 (15.4)	0.021
**Haematology**				
Haemoglobin (g/dL)	12.4 [10.4–14.2]	12.5 [10.5–14.2]	10.8 [9.6–13.3]	<0.001
Platelet count (×10^3^/µL)	273 [220–339]	274 [221–341]	269 [206–310]	0.014
Fibrinogen (mg/dL)	511 [394–696]	514 [398–697]	472 [366–628]	0.006
INR	0.98 [0.92–1.07]	0.98 [0.92–1.07]	1.00 [0.94–1.09]	0.067
**Kidney function**				
Blood urea (mg/dL)	66 [40–117]	66 [40–113]	97 [50–161]	<0.001
Serum creatinine (mg/dL)	1.60 [1.00–2.94]	1.60 [1.00–2.90]	2.03 [1.07–4.02]	0.002
eGFR (mL/min/1.73 m^2^)	49.0 [23.3–82.7]	49.7 [23.6–82.8]	37.0 [15.9–72.2]	0.003
**Biochemistry**				
Total protein (g/dL)	6.50 [5.50–7.30]	6.50 [5.50–7.30]	6.80 [6.05–7.50]	0.002
Serum albumin (g/dL)	3.64 [2.90–4.25]	3.64 [2.85–4.25]	3.67 [3.16–4.30]	0.129
**Urinalysis**				
24 h proteinuria (g/day)	2.30 [0.80–5.40]	2.40 [0.80–5.50]	1.95 [0.70–4.36]	0.054

Data are median [25th–75th percentile] or *n* (%). *p*-values from Mann–Whitney U test (continuous) or chi-squared test (categorical). eGFR by CKD-EPI.

**Table 2:** Observed and expected bleeding rates by Kaczmarek score.

Score	*n*	Observed (%)	Expected (%) *	O/E Ratio
0	886	21/886 (2.4)	1.2	1.98
1	1000	50/1000 (5.0)	3.2	1.56
2	657	46/657 (7.0)	6.7	1.05
3	286	21/286 (7.3)	11.6	0.63
4	231	21/231 (9.1)	17.4	0.52
5	21	3/21 (14.3)	19.2	0.74

Expected rates from Kaczmarek et al. native kidney biopsy cohort [4]. AUC = 0.624 (95% CI: 0.585–0.663) *.

**Table 3:** Univariate logistic regression for candidate predictors of major bleeding.

Variable	OR (95% CI)	*p*
Age (years)	1.00 (0.99–1.01)	0.861
Female sex	1.42 (1.03–1.95)	0.031
Charlson index	1.04 (0.97–1.12)	0.283
Hypertension	1.58 (1.09–2.27)	0.015
Diabetes mellitus	0.97 (0.64–1.48)	0.898
Obesity	0.78 (0.51–1.17)	0.227
Heart failure	2.27 (1.46–3.53)	<0.001
Ischaemic heart disease	1.31 (0.86–1.99)	0.214
Cerebrovascular disease	1.88 (1.11–3.18)	0.019
Peripheral vascular disease	0.60 (0.30–1.20)	0.148
Solid neoplasm	2.35 (1.26–4.38)	0.007
Liver disease	1.68 (1.08–2.62)	0.022
Anaemia	2.51 (1.79–3.50)	<0.001
Acute kidney injury	1.53 (1.06–2.19)	0.022
Thrombocytopenia	1.60 (0.85–3.03)	0.147
eGFR < 30 mL/min/1.73 m^2^	1.77 (1.28–2.44)	<0.001
Fibrinogen < 511 mg/dL	1.47 (1.06–2.04)	0.023
Haemoglobin (g/dL)	0.86 (0.80–0.92)	<0.001
Platelets (×10^3^/µL)	1.00 (1.00–1.00)	0.035
Fibrinogen (mg/dL)	1.00 (1.00–1.00)	0.006
INR	0.99 (0.90–1.10)	0.922
Serum albumin (g/dL)	1.19 (0.99–1.43)	0.071
Systolic BP (mmHg)	1.00 (1.00–1.01)	0.139
eGFR (continuous)	0.99 (0.99–1.00)	0.014

OR, odds ratio; CI, confidence interval.

**Table 4:** Multivariable logistic regression and derived bleeding risk score.

Variable	β	OR (95% CI)	*p*	Pts	Prev. (%)
Female sex	0.344	1.41 (0.98–2.04)	0.067	1	44.1
Anaemia	0.694	2.00 (1.31–3.06)	0.001	2	44.9
Heart failure	0.745	2.11 (1.20–3.70)	0.009	2	8.2
Solid neoplasm	0.785	2.19 (1.11–4.31)	0.023	2	3.5
Liver disease	0.373	1.45 (0.87–2.42)	0.152	1	10.1
Hypertension	0.380	1.46 (0.95–2.24)	0.081	1	66.4
eGFR < 30 mL/min/1.73 m^2^	0.092	1.10 (0.71–1.69)	0.676	1	32.0
Fibrinogen < 511 mg/dL	0.653	1.92 (1.32–2.81)	<0.001	2	49.8
**Maximum score**				**12**	

β coefficients from the full 16-predictor multivariable model (Supplementary Table S1). Points: β > 0.50 = 2; β ≤ 0.50 = 1. Score range: 0–12.

**Table 5:** Observed bleeding rate by new score level.

Score	*n* (% of Cohort)	Events	Rate (%)
0	131 (4.8)	2	1.5
1	294 (10.9)	3	1.0
2	344 (12.7)	9	2.6
3	581 (21.5)	22	3.8
4	498 (18.4)	28	5.6
5	365 (13.5)	31	8.5
6	258 (9.5)	21	8.1
7	151 (5.6)	12	7.9
8	48 (1.8)	8	16.7
9	27 (1.0)	8	29.6
10	7 (0.2)	0	0.0

**Table 6:** Diagnostic performance at selected cut-offs.

Cut-Off	Sensitivity	Specificity	PPV	NPV	Youden
≥2	96.5%	16.4%	6.1%	98.8%	0.129
≥3	90.3%	29.5%	6.7%	98.2%	0.198
≥4	75.0%	51.3%	8.0%	97.3%	0.263
≥5	55.6%	69.7%	9.3%	96.5%	0.252
≥6	34.0%	82.7%	10.0%	95.7%	0.167
≥7	19.4%	92.0%	12.0%	95.3%	0.114

Optimal cut-off by Youden index: ≥4 (Youden = 0.264). PPV, positive predictive value; NPV, negative predictive value.

**Table 7:** Comparative summary.

	Kaczmarek	New Score
Components	4 (max 5 pts)	8 (max 12 pts)
Includes fibrinogen	No	Yes
Includes renal function	AKI (ICD)	eGFR < 30 mL/min/1.73 m^2^
AUC (95% CI)	0.624 (0.585–0.663)	0.676 (0.631–0.717)
Optimism-corrected AUC	—	0.660
ΔAUC	Reference	+0.052 (*p* = 0.004)
Low-risk rate	2.4% (score 0)	1.8% (score 0–2)
High-risk rate	14.3% (score 5)	9.3% (score ≥5)

## Data Availability

The data underlying this article cannot be shared publicly due to institutional and national data protection regulations governing patient health information. De-identified data may be made available by the corresponding author (G.Ș.) upon reasonable request and subject to approval by the institutional ethics committee.

## Supplementary Materials

The following supporting information can be downloaded at: https://www.mdpi.com/article/10.3390/jcm15176515/s1, Table S1: Full multivariable binary logistic regression model with all 16 candidate predictors of major bleeding after percutaneous native kidney biopsy.
