# Supplementary material for: Predicting Major Bleeding in Native Kidney Biopsies: From External Validation of the Universal Bleeding Score to a New Clinical Tool
Source: J Clin Med. 2026 Aug 23;15(17):6515. doi: 10.3390/jcm15176515 (PMC13565800; doi:10.3390/jcm15176515)
Supplement: Supplementary file 1 [file jcm-15-06515-s001.zip › jcm-4445411-supplementary.pdf]

Supplementary Table S1. Full multivariable binary logistic regression model with all 16 candidate predictors of major bleeding after percutaneous native kidney biopsy.

Variable	β	SE	z	P-value	OR	95% CI	Retained
**Female sex**	**0.344**	**0.188**	**1.831**	**0.067**	**1.411**	**0.976–2.038**	**Yes**
**Hypertension**	**0.380**	**0.218**	**1.745**	**0.081**	**1.463**	**0.954–2.242**	**Yes**
Diabetes mellitus	−0.004	0.249	−0.016	0.987	0.996	0.611–1.622	No
Obesity	−0.125	0.236	−0.528	0.597	0.883	0.556–1.402	No
**Heart failure**	**0.745**	**0.287**	**2.596**	**0.009**	**2.107**	**1.200–3.699**	**Yes**
Ischaemic heart disease	−0.048	0.286	−0.167	0.868	0.953	0.544–1.671	No
Cerebrovascular disease	0.150	0.364	0.411	0.681	1.161	0.569–2.370	No
Peripheral vascular disease	−0.603	0.398	−1.517	0.129	0.547	0.251–1.193	No
**Solid neoplasm**	**0.785**	**0.345**	**2.274**	**0.023**	**2.192**	**1.114–4.311**	**Yes**
**Liver disease**	**0.373**	**0.261**	**1.433**	**0.152**	**1.452**	**0.872–2.420**	**Yes**
**Anaemia**	**0.694**	**0.216**	**3.209**	**0.001**	**2.001**	**1.310–3.057**	**Yes**
Thrombocytopenia	0.112	0.361	0.311	0.756	1.119	0.551–2.271	No
**eGFR <30 mL/min/1.73 m²**	**0.092**	**0.221**	**0.419**	**0.676**	**1.097**	**0.711–1.691**	**Yes**
Acute kidney injury	0.093	0.228	0.408	0.684	1.097	0.702–1.714	No
**Fibrinogen <511 mg/dL**	**0.653**	**0.193**	**3.375**	**<0.001**	**1.921**	**1.315–2.806**	**Yes**
INR >1.2	0.262	0.308	0.851	0.395	1.300	0.710–2.379	No

Model summary

Complete cases: n = 2,506 (132 major bleeding events, 5.27%); events per variable: 8.3.

Apparent AUC: 0.701; AIC: 1004.8; BIC: 1103.9.

Likelihood ratio test (vs. null model): P < 0.001.

Abbreviations: β, regression coefficient; SE, standard error; OR, odds ratio; CI, confidence interval; eGFR, estimated glomerular filtration rate (CKD-EPI); INR, international normalised ratio. Anaemia was defined as haemoglobin below sex-specific WHO thresholds (<13 g/dL in males, <12 g/dL in females). Thrombocytopenia was defined as platelets <150 ×10³/µL. Fibrinogen was dichotomised at the cohort median. Variables with P < 0.05 (anaemia, heart failure, solid neoplasm, fibrinogen <511 mg/dL) formed the core of the final 8-component score. Female sex, hypertension, liver disease and eGFR <30 were additionally retained based on consistent effect direction, clinical plausibility and established literature support [12–14]. Bold variable names indicate predictors retained in the final score.
